# Contralateral C7 nerve transfer through posterior vertebral approach combined with selective posterior rhizotomy of the affected cervical nerve in the treatment of central upper limb spastic paralysis: A case report

**DOI:** 10.1097/MD.0000000000025061

**Published:** 2021-03-26

**Authors:** Jingyu Guan, Jun Lin, Xueqing Guan, Qiang Jin, Wenchuan Zhang

**Affiliations:** a Departments of Neurosurgery, General Hospital of Northern Theater Command; b College of Medicine, China Medica University; c Departments of Anesthesiology, General Hospital of Northern Theater Command, Shenyang, Liaoning; d Department of Neurosurgery, The Ninth Hospital Affiliated to Shanghai Jiaotong University, Shanghai, China.

**Keywords:** C7 nerve, hemiplegia, selective posterior rhizotomy, transfer

## Abstract

**Introduction:**

C7 nerve transfer alone can improve upper limb motor function and partial spasticity. Selective posterior rhizotomy (SPR) of the cervical nerve alone can comprehensively improve spasticity but without neuromotor regeneration. We propose a novel possible improvement of contralateral C7 (CC7) nerve transfer through the posterior vertebral approach, which was combined with SPR of the affected cervical nerve.

**Patient Concerns:**

A 33-year-old male patient presented with cerebral hemorrhage of the left basal ganglia, paralysis of the right limbs, and hypesthesia 8 months earlier. The dysfunction of the affected hand was already present at admission. The patient reported a previous history of hypertension for several years and oral antihypertensive drugs, and blood pressure was controlled within a normal range.

**Diagnosis:**

Central upper limb spastic paralysis. The muscle strength of the right lower limb was grade IV. The Fugl-Meyer score of the right upper limb was 7 points, and the modified Ashworth score was 10.

**Interventions:**

The patient underwent CC7 transfer and SPR.

**Outcomes:**

The patient successfully underwent CC7 transfer and SPR without complications. On the day after surgery, the left upper limb motions were normal. The Fugl-Meyer score was 9, and the modified Ashworth score of the right upper limb was 2.

**Conclusions:**

CC7 nerve transfer through the posterior vertebral approach combined with SPR of the affected cervical nerve can possibly improve the surgical outcomes of selected patients with upper limb motor dysfunction and partial spasticity. This method has not been reported in the literature before, and additional studies are necessary.

## 1 Introduction

Professor Gu reported for the first time in 1992^[1]^ that the C7 nerve can be used as a source of nerves, without significant damage to the function of the contralateral side, and proposed the contralateral C7 (CC7) nerve transfer by the anterior vertebral approach. Later, Professor Xu applied, for the first time, CC7 nerve transfer by the anterior vertebral approach for the treatment of central upper limb spastic paralysis, leading to an improvement of the Fugl-Meyer score by 30 points.^[2–4]^

CC7 nerve transfer is an important treatment option for brachial plexus injury or central upper limb paralysis, and the anterior vertebral approach is mainly used (Xu's CC7 procedure).^[1–4]^ The present study explored the application of the posterior vertebral approach for CC7 transfer, in which the CC7 nerve is directly anastomosed to the C7 nerve of the affected side. The length of the transfer path is only about 4 cm (Guan's CC7 procedure). Thus, it avoids nerve transplantation, reduces the possible complications of vertebral artery injury, esophageal fistula, and upper limb pain during swallowing caused by the anterior vertebral approach.^[5]^ Selective posterior rhizotomy (SPR) of the cervical nerve alone can comprehensively improve spasticity but without neuromotor regeneration.^[6]^

Here, we propose a novel possible improvement of CC7 nerve transfer through the posterior vertebral approach, which was combined with selective posterior rhizotomy (SPR) of the affected cervical nerve. We report here 1 patient with central upper limb spastic paralysis who underwent this surgery.

## 2 Case presentation

A 33-year-old male patient presented with cerebral hemorrhage of the left basal ganglia, paralysis of the right limbs, and hypesthesia 8 months earlier. The dysfunction of the affected hand (reaching for objects, grasping movements, dressing, tying shoelaces, twisting a towel, or using a mobile phone) was already present at admission. No mental disorder was reported. The patient reported a previous history of hypertension for several years and oral antihypertensive drugs, and blood pressure was controlled within a normal range.

**Figure 1:**
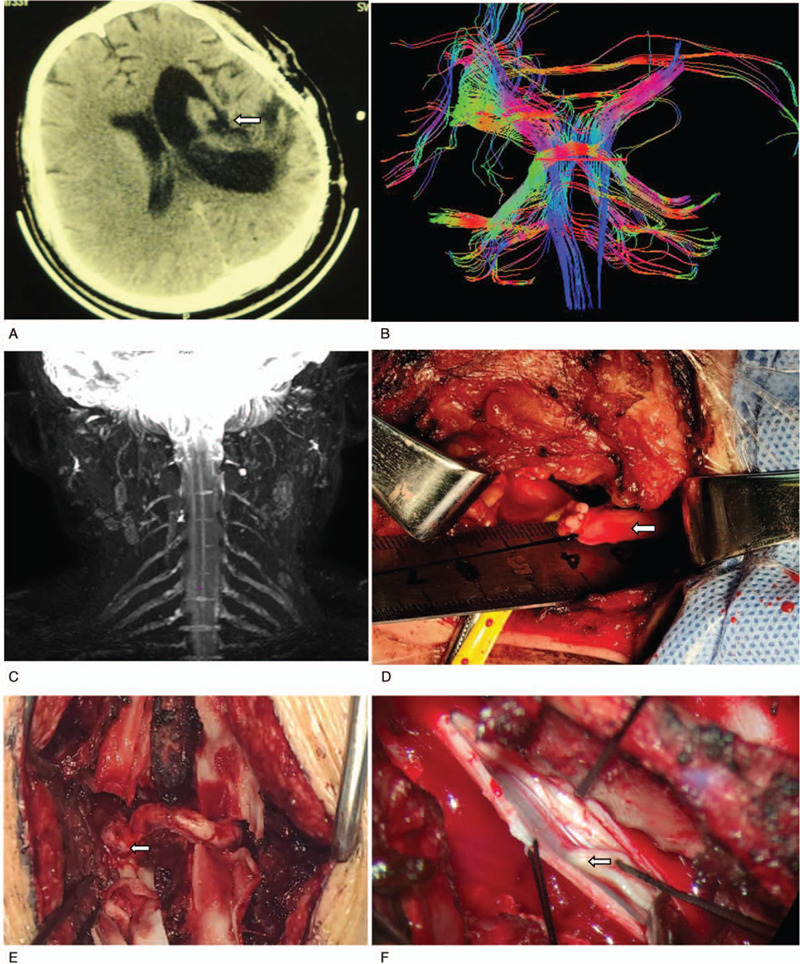
A: Preoperative head computed tomography (CT) scan revealing a softening lesion of the left basal ganglia. B: Diffusion tensor imaging (DTI) of the head showing a significant decrease in fibers in the left basal ganglia. C: Magnetic resonance imaging (MRI) scan of the brachial plexus, revealing normal bilateral brachial plexuses. D: Cutting and measuring the CC7 nerve. E: Tension-free anastomosis between the left C7 nerve and the right one. F: Exploring the posterior root of the C6 nerve.

Limb examinations showed that the muscle strength of the right lower limb was grade IV. The Fugl-Meyer score (a score used to determine the functions of the upper limbs after stroke; high scores indicate better outcomes; the maximum scores for upper extremities is 66^[7]^) of the right upper limb was 7 points, and the modified Ashworth score was 10 (elbow extension, 2; forearm rotation, 2; wrist extension, 2; thumb extension, 2; fingers 2–5 extension, 2) (the modified Ashworth score is an assessment of muscle spasticity in neurological conditions; low scores indicate better outcomes; the maximum score for each item is 2^[8]^). A preoperative computed tomography scan of the head suggested a softening lesion of the left basal ganglia (Fig. 1A). Head diffusion tensor imaging suggested a significant decrease in fibers in the left basal ganglia (Fig. 1B). Magnetic resonance imaging scan of the brachial plexus showed that the bilateral brachial plexuses were unremarkable (Fig. 1C).

Based on the low Fugl-Meyer score and the high modified Ashworth score, the patient was diagnosed with central spastic paralysis of the right upper limb. After evaluation by the surgeons, the patient agreed to undergo CC7 nerve transfer through the posterior vertebral approach combined with SPR of the affected cervical nerve.

## 3 Separation of the CC7 nerve

After general anesthesia, the patient underwent electrophysiological monitoring. He was in the supine position, and the left supraclavicular transverse incision was about 5 cm in length. The skin, subcutaneous tissues, and platysma were cut layer by layer. The skin flap was separated downward along the deep platysma to expose the external cervical triangle. The adipose tissues outside the brachial plexus were separated along the outer edge of the sternocleidomastoid muscle and the external jugular vein. The omohyoid was separated, and the transverse cervical artery was ligated or opened to expose the brachial plexus between the anterior and middle scalene muscles. The superior, middle, and inferior trunks of the left brachial plexus and phrenic nerve were separated and exposed. The phrenic nerve on the surface of the anterior scalene muscle was protected, and the anterior scalene muscle was cut. The C7 nerve root of the brachial plexus was exposed, and the proximal end was dissociated to the intervertebral foramen. The anterior and posterior divisions of the C7 nerve root were separated to the distal end to increase its length and then cut it off. At the same time, the length for the nerve root from the intervertebral foramen (marked by the anterior tubercle of the transverse process) to the subdivision (Fig. 1D) was measured. The incision was sutured.

## 4 CC7 nerve transfer and anastomosis

The patient was turned over in the prone position. A posterior median incision of the neck was made, and a median incision was made centered on the spinous processes of C6 and C7, about 10 cm in length. The skin, subcutaneous tissues, and ligamentum nuchae were cut. A periosteal dissector was used to perform subperiosteal dissection along both sides of the cervical spinous process. A vertebral retractor assisted in fully exposing the C6 and C7 spinous processes, vertebral plate, and zygapophysial joints. An osteotome was used to cut the superior and inferior articular processes on the left to expose and separate the left C7 intervertebral foramina, and the cut distal end was pulled out from the opened intervertebral foramina. The spinous processes of C6 and C7 were drilled near the vertebral plate to form an approximately 1-cm hole channel. The distal end of the left C7 nerve was penetrated from the hole channel, and the superior and inferior articular processes on the right were cut to expose and separate the right C7 nerve. The right C7 nerve in the middle of the intervertebral foramina was cut, and tension-free anastomosis with the left C7 nerve stump was performed (Fig. 1E). Bilateral superior and inferior articular processes were fixed.

## 5 SPR of the affected nerve

Drilling was performed to remove the right vertebral plates of C4–5 and T1-2, and the dura mater was cut. The posterior nerve roots of C5, C6, C8, and T1 were explored at the 4 intervertebral foramina of C4-5, C5-6, C7, T1, and T1-2 (Fig. 1F). Under electrophysiological monitoring, some of the posterior nerve roots were cut. After checking for the absence of active bleeding, the dura mater was sutured, and the neck muscles and skin were sutured layer by layer. A neck brace was used for fixation. The patient was sent to the ICU.

## 6 Follow-up

The next day after surgery, the patient was awake, and the tracheal cannula was removed. The left upper limb motions were normal. The patient received follow up during hospitalization. There was no operative complications, the incision healed well, and the suture was removed smoothly. At 1 day after and 2 weeks after operation, the Fugl-Meyer score was 9, and the modified Ashworth score of the right upper limb was 2 (elbow extension, 0; forearm rotation, 0; wrist extension, 1; thumb extension, 1; fingers 2–5 extension, 0) (Table 1). Up to now, 1 year after operation, muscle tension is the same as 2 weeks after operation. The patient had shoulder abduction and elbow flexion. These movements were not performed preoperatively.

**Table 1:** Evaluation of motor function damage and spasticity (muscle tension) of the affected upper limbs before and after surgery.

Paralyzed hand and evaluation	Prior to surgery	1 d after/2 wk after surgery
Side of paralyzed hand (left or right)	Right	Right
Total Fugl-Meyer score^∗^	7	9
Modified Ashworth Scale Score^∗∗^		
Elbow extension	2	0
Forearm rotation	2	0
Wrist extension	2	1
Thumb extension	2	1
Fingers 2–5 extension	2	0
Range of motion (°)		
Elbow	0	0
Forearm rotation	15	15
Wrist	0	0
Able to accomplish 3 or more functional tasks (yes/no)^#^	No	No

∗ High Fugl-Meyer scores indicate better results.

∗∗ Low Modified Ashworth Scale Score indicates better results.

# Able to accomplish 3 or more functional tasks: wearing clothes, tying shoelaces, twisting a towel, or using a mobile phone.

## 7 Discussion

CC7 transfer was first described in 1992^[1]^ and was later described using the anterior vertebral approach for the treatment of central upper limb spastic paralysis.^[2–4]^ CC7 transfer using the posterior approach was described in 2019^[5]^ and avoids the complications of the anterior approach. SPR of the cervical nerve alone can be used to improve spasticity without neuromotor regeneration,^[6,9–11]^ but it was not tried before in combination with CC7 transfer using the posterior approach.

In our clinical practice, an ideal case was encountered for such surgery. CC7 nerve transfer was completed through the posterior vertebral approach to the treatment of central upper limb paralysis. This approach needs a shorter nerve pathway than the anterior vertebral approach, and all patients do not need additional nerve transplantation.^[5]^ Regardless of the anterior or posterior vertebral approach, the main function of CC7 nerve transfer is to improve the extension and grasping function of the affected extensor muscle. At the same time, because the posterior nerve root of the affected C7 nerve is cut, it will partially improve the spasticity of the flexor.^[4]^

There are many treatment options for central limb spastic paralysis. Among them, SPR is the main surgical procedure.^[6,9–11]^ Given there are more posterior nerve roots of the cervical nerve that can be cut, the effect of improving spasticity is better than that of CC7 nerve transfer. Therefore, for spastic hemiplegia caused by stroke, the combination of CC7 nerve transfer and SPR of the affected side should achieve better clinical results. In the present report, CC7 nerve transfer through the posterior vertebral approach was combined with SPR of the affected side to treat central upper limb spastic paralysis. It not only achieved the main effects of CC7 nerve transfer in improving extension and grasping functions of the affected extensor muscle but also improved the limb spasticity. No related reports are available in the literature.

In addition, effective rehabilitation after CC7 transfer is an important measure for functional recovery, but sometimes limb spasticity will affect the implementation of rehabilitation treatment, such as manipulation and electrical stimulation.^[12]^ Thus, the combination with SPR of the affected cervical nerve will undoubtedly improve spasticity and promote rehabilitation. In the case reported here, long-term outcomes are not yet available. Nevertheless, so far, no complications were observed. Of course, the effectiveness and complications will have to be confirmed by future studies.

## 8 Conclusions

In conclusion, CC7 nerve transfer through the posterior vertebral approach combined with SPR of the affected cervical nerve can possibly improve the surgical outcomes of selected patients with upper limb motor dysfunction and partial spasticity. This method has not been reported in the literature before, and additional studies are necessary.

## Author contributions

**Conceptualization:** Jingyu Guan.

**Data curation:** Jun Lin, Xueqing Guan, Qiang Jin, Wenchuan Zhang.

**Formal analysis:** Jingyu Guan.

**Investigation:** Jun Lin, Xueqing Guan, Qiang Jin, Wenchuan Zhang.

**Methodology:** Jingyu Guan, Jun Lin, Xueqing Guan, Qiang Jin.

**Project administration:** Jingyu Guan.

**Writing – original draft:** Jingyu Guan, Jun Lin, Xueqing Guan, Qiang Jin, Wenchuan Zhang.

**Writing – review & editing:** Jingyu Guan.
